# Meeting report: Advancing accelerated regulatory review with Real-Time Oncology Review (RTOR), Project Orbis, and the Product Quality Assessment Aid

**DOI:** 10.1186/s41120-022-00066-1

**Published:** 2022-12-05

**Authors:** Marquerita Algorri, Ajay Acharya, James Bernstein, Nina S. Cauchon, Xiao Hong Chen, Kim Huynh-Ba, Carol Krantz, Tao Li, Yiwei Li, Sherita McLamore, Scott W. Roberts, David Schwinke, Rakhi Shah, Andrea Schirmer, Helen Strickland, Kin Tang, Timothy Watson

**Affiliations:** 1 Amgen Inc., Thousand Oaks, CA USA; 2 Merck & Co., Inc., Kenilworth, NJ USA; 3 Live Oak Pharmaceutical Consulting, Inc., Raleigh, NC USA; 4 U.S. Food and Drug Administration, Office of Pharmaceutical Quality - Office of New Drug Products, Silver Spring, MD USA; 5 Pharmalytik, LLC, Newark, DE USA; 6 Seagen, Inc., Bothell, WA USA; 7 U.S. Food and Drug Administration, Office of Pharmaceutical Quality - Office of Pharmaceutical Manufacturing Assessment, Silver Spring, MD USA; 8 Novo Nordisk A/S, Vandtårnsvej 114, DK-2860 Søborg, Denmark; 9 AbbVie, North Chicago, IL USA; 10 Boehringer Ingelheim Pharmaceuticals, Inc., Ridgefield, CT USA; 11 GlaxoSmithKline US, Research Triangle Park, NC USA; 12 Genentech, Inc., South San Francisco, CA USA; 13 Pfizer Inc., Groton, CT USA

**Keywords:** Regulatory, FDA, Regulatory efficiency, Accelerated review, Chemistry, Manufacturing, and Controls, Project Orbis, Product Quality Assessment Aid, Real-Time Oncology Review

## Abstract

The American Association of Pharmaceutical Scientists (AAPS) Chemistry, Manufacturing, and Controls (CMC) Community hosted two virtual panel discussions focusing on several novel regulatory review pathways for innovative oncology products: Real-Time Oncology Review (RTOR), Project Orbis, and the Product Quality Assessment Aid (PQAAid). The panel sessions were held on August 27, 2021, for the discussion of RTOR, and January 21, 2022, for the discussion of Project Orbis and the PQAAid. Both panel sessions included representatives from the US Food and Drug Administration (FDA) and subject matter experts from the pharmaceutical and biotechnology industries, with the aim of facilitating knowledge sharing on CMC-specific advantages, challenges, eligibility criteria for participation, and operational modifications instituted through the utilization of these acceleration initiatives. Key topics included managing cross-regional regulatory CMC requirements, adapting to expedited development timelines, coordinating interactions between health authorities and industry, and potential opportunities for future improvement and expansion of these programs. As RTOR, Project Orbis, and PQAAid are relatively new initiatives, the experiences shared by the panel experts are valuable for providing deeper insight into these new regulatory pathways and processes.

## Introduction

Patients with critical illnesses around the world rely on timely access to safe and effective therapeutics. For many complex and serious disease states, patients are adversely impacted by long development timelines that impede the availability of new drugs. Within the past decade, clinical development processes for innovator products have taken an average of 9.1 years to advance from initial clinical development to marketing approval in the USA (Brown et al. 2021). Efforts have been made, globally, by the pharmaceutical industry and health authorities to reduce drug development timelines to expedite access to new drugs. Accordingly, reductions in drug development and approval timelines have been shown to create substantial improvements in patient lifespan and quality of life (Pharmaceutical Research and Manufacturers of America (PhRMA) 2022).

While time-sparing regulatory enablers for expedited approvals, such as Accelerated Approval, Fast Track, Breakthrough Designation, and Priority Review, have been in use in the USA for almost 30 years, within the past several years, the Food and Drug Administration (FDA) has leveraged the Oncology Center of Excellence (OCE) to establish new, unique regulatory acceleration and collaboration programs aimed at promoting regulatory efficiency (Kepplinger 2015; US Food and Drug Administration 2014). OCE was introduced in 2017 under the 21st Century Cures Act, with the goal of progressing and expediting the development and approval of cancer therapeutics (US Food and Drug Administration 2022a). Several of OCE’s initiatives aim to transform the traditional regulatory review and approval paradigm by providing new instruments and methodologies to facilitate submission and review for oncology products of paramount clinical significance. Real-Time Oncology Review (RTOR), Project Orbis, and the Assessment Aid (AAid) are the OCE initiatives highlighted in this meeting report.

RTOR, Project Orbis, and the AAid serve to further accelerate review, and in the case of Project Orbis, potentially result in streamlined reviews of applications to multiple countries. Due to the relative novelty of these programs, many open questions remain within the regulatory ecosystem regarding the pathways’ utility, applicability to specific application types, impact on timelines, and regional considerations. To promote supportive knowledge sharing across industry and FDA, two AAPS CMC Community Virtual Panel Discussions were organized to facilitate discussion on prior experiences and future opportunities for RTOR, Project Orbis, and the role of the AAid. While the sessions were broadly scoped for discussions pertaining to small molecule drugs, many of the topics and perspectives presented are also relevant to other modalities.

This meeting report provides a consolidated summary of discussions adapted from two AAPS Chemistry, Manufacturing, and Controls (CMC) Community Virtual Panel Discussions focusing on RTOR and Project Orbis, held on August 27, 2021, and January 21, 2022, respectively. Both sessions featured speaker panels comprised of industry and FDA expert speakers who provided insights into their perspectives on the benefits and challenges of these new regulatory initiatives.

## RTOR virtual panel sesssion: August 27, 2021

### Virtual panel introduction and presentation: RTOR

Expert panelists, Xiao Hong Chen (US FDA) and Yiwei Li (US FDA), provided an overview of the RTOR program, its applicability to CMC data, and the involvement of the Product Quality Review Team. RTOR began as a pilot program in 2018, with the aim of facilitating early submission of key safety and efficacy data for oncology programs to allow regulators to initiate the review sooner than typically possible, particularly for cancer therapeutics of clinical significance that represent an improvement to the standard of care.

Under the current regulatory submission and review paradigm, sponsors submit a complete regulatory dossier containing all required modules and data once all materials are available. However, assembling a major regulatory filing often requires several months for sponsors to collate and finalize all components due to the extensive quantity of data and information required. The investigational drugs under the current “Fast Track” program are allowed to be submitted containing individual modules in a rolling submission. However, each submitted module should contain all required information. Using RTOR, regulatory documents can be submitted asynchronously in waves, as they are finalized, in “split” or partial modules. This change in submission cadence enables FDA reviewers to initiate the review process at an earlier timepoint and to distribute the workload more efficiently over time.

While the RTOR pilot was originally positioned to include only clinical data to support efficacy supplements, the program later expanded to support applications for new molecular entities, including CMC data. As of July 2021, there were 29 supplements and 10 new molecular entity (NME) applications approved using RTOR. Participation in RTOR is conducted on a voluntary basis and can be initiated by either sponsor or FDA request. Eligibility and acceptance are based on clinical considerations and discussion between the sponsor and Agency. Inclusion in the RTOR program does not influence or guarantee approval or impact upon federally endorsed review timelines as described in the most current version of the Prescription Drug User Fee Act (PDUFA).

Prior to submission, sponsors must meet with the Agency, including the Office of Pharmaceutical Quality (OPQ), to discuss and agree on the submission plan to ensure a logical and efficient submission and review schedule. For example, Module 3 sections that should be reviewed together are scheduled accordingly for simultaneous submission. While companies have used variable approaches due to product, modality, and situational differences that impact the submission strategy, general recommendations from FDA suggest that clinical data should be submitted 7–10 weeks early, with CMC information to follow at a later time. However, proactive discussion with the Agency on the submission schedule is vital, as companies have been able to leverage different approaches for submitting CMC data on a rolling basis. CMC data can be submitted in “waves”:

- *Wave 1 (CMC)*: Manufacturing process information; drug substance, drug product, and critical intermediate manufacturing and testing facilities with identification details (address, FDA Establishment Identification (FEI) number) and responsibilities. Facility information should be prioritized because facility assessments are complex and require significant planning and coordination.
- *Wave 2 (CMC)*: Stability data, which relies on real-time results and hence is often on the critical path, can be submitted in wave 2. This wave should include any other outstanding information needed to complete the application.

Expert industry panelist Carol Krantz (Seagen) shared published data from February 2018 through April 2020 demonstrating the ability of RTOR to substantially reduce review and approval timelines. In comparison to Priority Review, another accelerated review mechanism used by FDA in which the review timeline is shortened to facilitate an action date within 6 months, RTOR enabled shorter median approval times for new molecular entities (2.2-month reduction) and supplements (2.7-month reduction), wherein 90% of applications using RTOR were actioned prior to the PDUFA date (de Claro et al. 2021).

### RTOR panel discussion

Following the RTOR introduction, the FDA and industry expert panelists participated in a question-and-answer session. The panelists included Xiao Hong Chen (US FDA), Yiwei Li (US FDA), Carol Krantz (Seagen), and Timothy Watson (Pfizer). Nina Cauchon and James Bernstein moderated the panel discussion. Kim Huynh-Ba and Helen Strickland captured meeting minutes. Kin Tang was the AAPS lead organizer of the event and Scott Roberts provided meeting support. The key topic areas for the panel session included:

1. Experiences using RTOR for CMC
2. Similarities and differences between RTOR and other accelerated programs
3. Opportunities for enhancing CMC workflow efficiency
4. Delay-causing factors for providing CMC information for RTOR
5. Challenges and opportunities for improvement

#### Knowledge sharing through questions

**Please share your experiences with RTOR or other relevant applications from the perspective of CMC**

Carol Krantz shared her experiences with Seagen’s product, TUKYSA® (tucatinib). While Seagen was not originally intending to pursue accelerated review for this product, upon receipt of the unblinded clinical study data, FDA invited their participation in RTOR and Project Orbis pilot programs and to use the AAid and Product Quality Assessment Aid (PQAAid) templates for clinical/nonclinical and CMC information, respectively. Upon Seagen’s acceptance, FDA and Seagen developed the overall submission strategy collaboratively.

Throughout the submission and review process, communication between the sponsor and FDA is pivotal. During the review, Seagen worked closely with the FDA Quality Review team to discuss modifications in the submission plan as well as potential roadblocks. On the Agency’s side, FDA emphasized the importance of early interaction between cross-functional review team members, which enables critical issues to be identified and addressed more readily. Under RTOR, the Clinical Reviewer and the Quality Reviewer initiate their collaboration early in the review process, which is not typical for standard reviewing procedures as typically the Quality Reviewer joins the review team at a later stage.

For the TUKYSA® New Drug Application (NDA), the first wave of submission activities occurred in November 2019, with the remaining sections submitted in December 2019. For RTOR filings, wave 1 includes the majority of the clinical data; however, the full CMC data package may not be available at this time. Components such as executed batch records, including complete translations from foreign manufacturing facilities, may not be available until a later time. The CMC development strategy should work in unison with the clinical development plan to ensure optimal efficiency when pursuing accelerated submission pathways.

Seagen experienced difficulties adjusting to the dramatic acceleration in timelines, which were further complicated by technical challenges late in the product’s development. Despite these complexities, through efficient teamwork and open communication between Seagen and FDA, TUKYSA® gained approval within 120 days of full NDA submission.

**What are the learnings from other expedited programs that can be applied to RTOR?**

Several accelerated review programs predate the advent of RTOR, which has enabled sponsor companies and the FDA to advance their tools and strategies for navigating a dynamic submission and review paradigm. Quality by design (QbD), as outlined in ICH Q8, is a core concept in pharmaceutical development which emphasizes the establishment of pre-defined objectives that promote process and product control and understanding (Yu et al. 2014). QbD is crucial for success in an expedited environment because it empowers sponsors to obtain more knowledge on their product and processes firsthand, which allows for greater agility in preparing responses to questions and critically assessing manufacturing processes throughout the review period.

The principal challenge manufacturers face in an accelerated development scenario occurs during the formal transition from clinical development to the commercial phase. Under accelerated conditions, the commercial process must be designed and finalized within an abbreviated time period, which means that decisions such as optimal formulation, synthetic process, and fit-for-purpose manufacturing and controls must be decided upon early. To support cross-functional timeline alignment, companies should develop strategies for commercial processes and facilities in parallel to clinical development. Early-stage planning will enable companies to build up and accumulate knowledge of their product in advance of the planned filing to reduce the risk of CMC-mediated delay. Towards this goal, the CMC submission strategy should be initiated as soon as clinical data are available and agreed upon with FDA. To support acceleration more broadly across product areas and modalities, sponsors can consider changing the product development paradigm by transitioning from a stepwise, gated process to an approach utilizing team triage to responsively address patient needs.

The expert panelists emphasized that communication with the Agency is integral to ensure alignment on the strategy. Conducting a CMC-focused meeting between sponsor companies and FDA prior to submission can help to confirm and adapt the strategy. Initiating interactions with the Agency at the right time can help to support a cohesive accelerated strategy. The meeting should be held at a stage of process development in which the sponsor has enough information to support and inform their strategy, but there is sufficient time to initiate changes based on the Agency’s feedback.

In situations wherein a company wishes to pursue Emergency Use Authorization (EUA), there are many junctures at which acceleration can be further enhanced. For example, an EUA and an Investigational New Drug Application (IND) can be active at the same time, wherein the EUA builds upon the information provided in the IND. A similar concept could theoretically be adapted for RTOR, wherein an NDA/Biologics License Application (BLA) uses the IND as a foundation, which can be updated as development progresses.

Similarly, the early development and engagement model utilized for RTOR could be considered for non-RTOR filings for incorporation into the overall CMC development strategy to support early action and timely development even outside of the context of oncology products. For example, for advice on the acceptability of a given test method, such as the dissolution assay, sponsors may submit supporting information directly to the IND, in advance of the NDA filing, to request a review on the suitability of the test method for commercial use.

**What workflow opportunities for enhancing the effectiveness of delivery and review of CMC information have been identified based on RTOR?**

While each company may have different internal processes or approaches for CMC development and submission assembly, the RTOR submission strategy should be based on the feasibility and timing of content delivery. Early and focused communication between FDA and sponsors remains significant throughout the submission and review process. When utilizing innovative approaches, the industry should be open to engaging in scientific dialogue with the Agency in collaborative problem solving.

In regard to specific submission components, stability, facilities information, and validation data are often lagging behind other datasets due to logistical aspects and extended data collection timelines. For example, ICH guidance requires 12 months of stability data to be provided at the time of NDA/BLA filing, but for accelerated filings, this information may not be available at the time of initial submission due to compressed development timelines. In this scenario, the sponsor may be able to discuss with FDA the possibility of submitting partial stability information and providing an updated data package during the review. In some circumstances, it may be possible, pending Agency agreement, to provide supporting stability data if batches are considered to be representative.

Validation data, like stability data, also presents challenges for accelerated programs, as process validation typically occurs at a late stage of CMC development and may not be complete at the time of filing. To solve for timeline constraints, potential strategies include performing certain confirmatory studies post-approval, concurrent release of batches to allow for quick distribution following approval, and use of prior knowledge obtained during clinical and registrational batch processing. As previously indicated, nontraditional strategies should be discussed and agreed upon with FDA. The FDA Process Validation: General Principles and Practices guidance document provides insight on the contexts and circumstances in which concurrent release may be appropriate (US Food and Drug Administration 2011).

**What are the bottlenecks or delay causing factors for the first CMC interaction or even the submission of CMC information to an RTOR filing?**

Changes, whether planned or unexpected, can significantly impact the CMC development plan, filing strategy, and submission schedule. Additionally, the assessment of the adequacy of bridging strategy and data in the transition from clinical to commercial material remains a challenge for CMC filings. As discussed previously, communication between sponsors and FDA throughout development is key. When modifications are made, it is critical to notify the Agency within a timely manner to prompt discussion towards resolution and risk management.

**What are the challenges and opportunities for improvement that can be extended from RTOR?**

The goal and vision of expeditiously delivering safe and effective medicines to patients with serious conditions with unmet medical need is a driving factor across industry and FDA which inspires change and continual pursuit of improvement. To facilitate further advancement, the panelists suggested several opportunities for enhancing acceleration.

1. The use of clinical-stage CMC data—The information obtained from clinical lot production can be used to meaningfully inform and predict qualities of commercial lot production. For example, per ICH Q1A Guidance, stability information obtained from clinical lots may be representative of the proposed commercial product and may be suitable for inclusion in the NDA/BLA data package if commercial stability data is not yet available. This can help to ease the transition from clinical to commercial and opens the possibility for an initial launch from a clinical facility. FDA indicated that different tools and initiatives should be considered and noted that they are working on additional RTOR templates and programs aimed at streamlining review.
2. Use of concurrent release and validation—Process validation can contribute to significant delays. Concurrent release is permitted in specific circumstances, such as for the manufacture of orphan drugs, which can allow for continued access to therapy for patients with rare diseases who respond well to treatment, given the reasonable expectation that the manufacturing process is robust. Additional clarity and guidance are needed to establish the framework for how traditional programs can convert to accelerated programs using RTOR. Communication with the Agency can help to guide and inform sponsors’ validation approaches.
3. Use of predictive stability models for setting initial shelf life—In scenarios where there is limited long-term stability data, predictive tools such as the Accelerated Stability Assessment Program, which can be conducted in days to weeks, can help to improve understanding of the product’s stability characteristics. Application of predictive modeling has been increasingly used in IND filings. FDA has assembled an expert working group on stability models to help support regulatory decision-making on product shelf life.
4. Harmonization amongst agencies—Many companies coordinate concurrent global submissions to different agencies for major filings. Across regions, ICH Q8 and Q11 expectations are not uniformly interpreted, resulting in regional variability and challenges in managing global commercialization. The Agency recommended establishing a risk-benefit assessment tool to assess the breadth and scope of CMC-related data risks once clinical data are available.

## Project Orbis virtual panel session: January 21, 2022

### Project Orbis—virtual panel introduction and presentation

Following the RTOR panel discussion, a separate, but related panel discussion was conducted on Project Orbis, a collaborative application review process between partnering health authorities, featuring a different group of Industry and FDA expert panelists who shared their experiences and knowledge on Project Orbis and the AAid. Project Orbis and the AAid are regulatory efficiency-enabling programs also established by OCE, which may be used together to improve and accelerate submission and review tasks. Approximately 71% of applications that utilized Project Orbis and AAid also utilized RTOR, which demonstrates the synergy between these innovative approaches to regulatory submission and review.

Project Orbis is a multi-region collaborative review paradigm for oncology products of substantial clinical significance, which is intended to allow patients to obtain earlier access to therapeutics in participating countries (US Food and Drug Administration 2022a; de Claro et al. 2020). The AAid is a cross-functional review document that is populated by both the sponsor and health authority, including sponsor-initiated sections for data and supporting summaries, as well as regulator-specific sections for health authority assessment (US Food and Drug Administration 2021). The AAid aims to facilitate critical assessment of key information and enhance consistency between reviews. The PQAAid is an OPQ-specific iteration of the AAid which summarizes relevant quality information to assist regulator reviewing tasks for NDA/BLA original applications. In the context of Project Orbis, the AAid and PQAAid provide a medium for sharing applicant data summaries and reviewer assessments across regulatory organizations.

At the start of the session, expert panelists, Sherita McLamore (US FDA) and Rakhi Shah (US FDA), provided an overview of the Project Orbis program, including eligibility criteria, participating countries, and review processes. Project Orbis is led by FDA, but enables participating regulatory authorities, known as Project Orbis Partners (POPs), to review sponsor application materials contemporaneously or in a staggered fashion. Currently, participating POPs include the Australian Therapeutics Goods Administration (TGA), Brazil’s National Health Surveillance Agency (ANVISA), Health Canada, Israel Ministry of Health Pharmaceutical Administration, Singapore Health Sciences Authority (HSA), Swissmedic (Switzerland), and United Kingdom Medicines and Healthcare Products Regulatory Agency (MHRA—Great Britain) (US Food and Drug Administration 2022b). Though the regulatory review is conducted in a collaborative manner, which allows for sharing of materials such as information requests, FDA and POPs are responsible for directing their own independent reviews and ultimately will provide separate regulatory decisions. In addition to allowing for direct collaboration, Project Orbis also increases understanding of global regulatory processes amongst FDA and other regulators.

Similar to RTOR, participation in Project Orbis is voluntary and can be initiated by sponsors or FDA (Narayan et al. 2020). Eligible applications will be for original applications or efficacy supplements for oncology products which offer clinically significant improvements over the current standard of care, which generally also qualify for Priority Review. Once an application of interest is identified by either the sponsor or FDA, the sponsor must submit an application containing a list of POP countries requested for participation, authorization letters for each country which permit information sharing across Orbis countries, a submission plan including the submission schedule by country, and contact information for sponsor representatives. The FDA then shares the submission plan and key results from supporting clinical studies with the requested POPs to confirm their ability to participate in the review.

Once the submission plan and POP engagement are confirmed, participating POPs are required to sign a confidentiality agreement and must agree to participate in a minimum of 5 cross-agency POP meetings for original applications, or at least 3 POP meetings for supplements. While FDA, as the lead facilitator, is always included in every Orbis filing, not all POPs will be able to take part in every Orbis review process depending upon the Applicant’s list of proposed POPs and/or the willingness of a POP to participate depending on resources and timelines. Accordingly, reviews may consist of a select subset of POPs depending on resource availability.

Project Orbis includes three types of submission and review processes: type A, type B, and type C:

- Type A describes a submission and review framework wherein concurrent submission of the application occurs for all participating countries, enabling sharing of review materials, simultaneous review, and concurrent regulatory action may be possible.
- Type B is a modified process wherein review may or may not occur in parallel across countries. While FDA review reports are shared and multi-country meetings are held between FDA and POPs, it is generally not possible to have concurrent action with FDA.
- Type C submissions can be considered for applications for which FDA has already confirmed a regulatory decision. Collaboration is conducted via written-only interactions with no meetings between FDA and POPs. As FDA has already taken action, concurrent review or action is not possible.

Since the program’s inception, most Project Orbis applications have pursued review under the type A pathway, encompassing 72% of applications submitted from June 2019 through June 2020 (Narayan et al. 2020).

Industry expert panelist Tao Li shared his experiences with Project Orbis during the initial approval of Seagen’s product, TUKYSA®, which also utilized the RTOR program, as previously discussed. Notably, TUKYSA®, which gained approval in April 2020, was the first NME to be approved through Project Orbis. Seagen submitted applications to 5 POPs (FDA, TGA, HSA, Swissmedic, Health Canada) almost simultaneously, with all submissions occurring within a 30-day period. While some information requests were routed through FDA, others were received directly from other POPs. Overall, the collaborative approach enabled by Project Orbis facilitated harmonized yet independent review processes that yielded significant benefits, including reduction in overall review times as well as increasing Seagen’s efficiency for addressing regional differences across the participating POPs. The NDA approval was received in the US 4 months after the initial filing. Approvals were received in POP regions POPs within 4 months of the US approval, representing an average of 50.8% reduction in the review timeline across POP regions.

Since its establishment in May 2019, Project Orbis has continued to evolve and adapt new ways of working. When Seagen’s NDA was under review, Project Orbis did not yet include different reviewing categories, such as type A, type B, and type C. To provide perspective on a more current version of the Project Orbis process, industry panelist Ajay Acharya presented his experiences with Merck’s product, WELIREG® (belzutifan). WELIREG® was the sixth NME to be approved under Project Orbis in August 2021. Reviewing POPs included FDA, TGA, Health Canada, and MHRA, whereas a different regulatory review pathway for orphan diseases was pursued in Brazil. Submissions were staggered throughout 5 months after the initial FDA filing, enabling the use of Project Orbis type A and type B processes. Information requests from POPs were not routed through FDA, but Merck noted that they did not receive duplicative or similar queries across POPs if a response had already been provided. In examining the feedback received, there was overall alignment across POPs, which suggests the effects of transparency and information sharing within Orbis. FDA approval was received in January 2022, 1 month ahead of the PDUFA date. At the time of the virtual panel discussion in January 2022, applications in other regions were still undergoing review.

### Project Orbis panel discussion

Similar to the RTOR session, a question-and-answer session with the FDA and industry expert panelists was held following the introductory presentation. The expert panelists included Sherita McLamore (US Food and Drug Administration), Rakhi Shah (US Food and Drug Administration), Ajay Acharya (Merck) and Tao Li (Seagen). Nina Cauchon and Kin Tang moderated the panel discussion. Andrea Schirmer and Helen Strickland captured meeting minutes. Rita Algorri was the logistical coordinator of the event. Kim Huynh-Ba and David Schwinke provided organizational and logistical meeting support. The key topic areas for the panel session included:

1. CMC-focused experiences participating in Project Orbis and PQAAid
2. Role of the PQAAid in supporting Project Orbis
3. Scope of Project Orbis
4. Timeline management
5. Future opportunities for enhancing collaboration

#### Knowledge sharing through questions

**Please share your experiences, from the perspective of CMC, with using Project Orbis to collaborate and interact with other Agencies**

FDA expert panelist Sherita McLamore shared that the Agency has had overall positive experiences using Project Orbis as a facilitatory mechanism for interacting with other Agencies, gaining further knowledge on regulatory processes used in other regions, and engaging diverse perspectives through scientific dialogue. While there are challenges, such as time zone-related complexities in conducting cross-regional POP meetings and logistical challenges when applications are not submitted concurrently, ultimately, the insights gained from collaborative meetings and information requests provided by other regulators are useful and support FDA’s review processes.

From an industry perspective, Tao Li highlighted the time-saving benefits that Seagen attained as part of the concurrent submission, as all approvals from POPs were received in advance of standard timelines. In addition, there was also increased regulatory alignment across POPs, which reduced the burden of regional differences. As a specific example, most of the specification limits were aligned across POPs. Ajay Acharya noted the utility of regulator collaboration for managing staggered submissions, which helped to reduce the volume of follow-up questions and information requests from POPs. While using a simultaneous approach may have yielded a more consolidated set of questions, business needs and objectives vary, so the option to use different modes of collaborative review under Project Orbis can confer valuable flexibility. Sponsors must consider the different benefits and application-specific variables when selecting the regulatory submission strategy under Project Orbis.

**What is the Product Quality Assessment Aid, how is it used within Project Orbis, and what are the benefits and challenges associated with its use? Is it used for post-approval submissions?**

Rakhi Shah presented a blank PQAAid template and provided a brief overview of the document’s organization, which is subdivided into discrete sections to facilitate efficient review across disciplines within OPQ, including drug substance, drug product, manufacturing and facilities, and biopharmaceutics. The PQAAid was developed as part of the Project Orbis program and completion of the PQAAid within 30 days of the initial submission is a requirement for all original applications reviewed under Project Orbis. While no supplements reviewed through Project Orbis to date have contained CMC information, it can be envisioned that the PQAAid may also be useful for reviewing applicable supplements.

Sponsors can utilize the PQAAid to concisely summarize the CMC data package, including their position on key CMC topics. This can aid in further streamlining CMC reviews by providing a centralized location for reviewers to access important information outside of Module 3. During the review process, FDA reviewers within the four aforementioned disciplines are expected to populate the PQAAid with their findings, including risk assessments and deficiencies. The PQAAid usually spans approximately 80 pages as a template, which typically expands to 150 pages once all reviews are complete.

While the benefits of the PQAAid include streamlining communication across disciplines and POPs and providing a consolidated quality summary that all functions can view, challenges occur when FDA’s internal disciplines utilize division-specific or initiative-specific templates. Similarly, if POP reviews are occurring asynchronously, there is potentially less opportunity for collaboration using the PQAAid. Despite these potential challenges, both FDA and industry have found the PQAAid to be beneficial for enabling efficient review.

**Is the same CMC dossier submitted to all POPs? Were there variations in control strategies?**

There is no obligation to submit identical CMC dossiers to all participating POPs. The first section of the PQAAid addresses regional variations by requesting sponsors to identify and summarize key differences at a high level. Differences in packaging, specifications, and nomenclature are expected. While there is no formal requirement to submit similar data packages, it is beneficial for sponsors to limit differences across regional dossiers to avoid creating downstream complexities during submission management, collaborative review, and post-approval lifecycle management.

Industry expert panelists Ajay Acharya and Tao Li noted that while there was some divergence in the control strategy across regions for their respective programs, collaboration and alignment between POPs generally supported a strong core dossier. Differing opinions were noted for particular circumstances, such as the use of near-infrared spectroscopy.

**How are manufacturing site inspections managed? Are results of pre-approval inspections shared or are the inspections coordinated?**

Inspections are managed independently, as some countries do not require pre-approval inspections. Additionally, review and inspection timelines may significantly differ across agencies, creating challenges for meaningful coordination. While the inspection reports are not shared amongst POPs, inspection outcomes are communicated. In the future, there could be opportunities for collaboration that leverage other harmonization programs that FDA participates in.

**What is the scope of Project Orbis? Does it apply to small and large molecules? Will other countries and/or therapeutic areas be added?**

Project Orbis can be used to review CMC information included in applications for both small and large molecules. As part of the program’s continued evolution, more complex applications are being considered, including diagnostic devices and advanced therapy products. When considering expansion to other regions, some limitations exist due to local regulatory processes. For example, the European Medicines Agency (EMA) stops the review clock when deficiencies are identified, but FDA does not. As Project Orbis is an initiative of OCE, other therapeutic areas have not been considered as these are out of scope and would need to be managed under their corresponding divisions.

**What were the challenges relating to internal or external timeline management? How were the complexities of cross-regional timelines addressed?**

Managing accelerated timelines is often difficult for both regulators and sponsors. From the Agency’s perspective, the Office of Pharmaceutical Manufacturing Assessment (OPMA) faces a particularly substantial burden due to challenges associated with scheduling and conducting inspections, which were further exacerbated during the COVID-19 pandemic as a result of international travel restrictions. Project Orbis is uniquely resource-intensive due to the need for additional meetings conducted with POPs, which span several time zones and require scheduling flexibility from participants. One of the first NDAs to be reviewed under Project Orbis required 21 meetings involving CMC. With experience and business process changes, efficiency has increased over time, requiring less meetings.

Sponsors can utilize techniques such as staggering global submissions, or using other innovative review programs, such as RTOR, to help coordinate timelines. If global submissions are staggered, a core or baseline dossier can initially be developed and submitted for an initial region. The core dossier can then be modified based on region-specific requirements which may require additional time. If initiatives such as RTOR are used, facility information can be submitted in advance of the full dossier to facilitate early inspection.

**What opportunities do the Agency and industry see for further enhancing collaboration?**

Project Orbis has been greatly beneficial for patients by allowing products to reach multiple markets sooner. Type A and type B Project Orbis submissions that utilize a concurrent submission model have demonstrated the most success for reducing timelines. Opportunities that allow for increased alignment of the review process will further extend the benefits realized through Project Orbis.

## Conclusions

There is broad recognition across the pharmaceutical ecosystem that drug development and regulatory timelines must accelerate beyond their traditional pace to effectively address unmet medical needs in the context of serious and life-threatening illnesses. In the USA, 1 life-year is lost for every 2.2 min of delay in drug approval for oncology indications (Helwick 2015). Regulators and industry must make meaningful strides towards implementing and advancing innovative programs with demonstrated efficiency gains to benefit patients. As shown by successes gained through FDA’s RTOR and Project Orbis, regulatory collaboration is an integral enabler for acceleration. Collectively, these initiatives enable direct and proactive communication between sponsors and regulators, as well as across global regulators. While RTOR supports a modular, sequential format for data sharing as a more efficient mode of information exchange, Project Orbis offers the unique opportunity to drive towards cross-regional alignment through information sharing and scientific dialogue.

The diverse real-world experiences shared by the expert panelists during the AAPS panel discussions illustrated the many tangible benefits of OCE’s regulatory efficiency initiatives. Despite the challenges of navigating the uncertainties of participating in a new review paradigm and orchestrating abbreviated submission timelines, which can be particularly challenging for CMC applications which can have data requirements with extended timelines, sponsors and regulators emerged with overall positive perspectives and successes using RTOR, Project Orbis, and the PQAAid. Future opportunities that expand on these capabilities, their scope, and applicability across modalities and therapeutic areas will offer significant promise in transforming and reducing drug development timelines.

## Data Availability

Information in this article is summarized from discussions presented at the panel sessions. Data sharing is not applicable to this article as no datasets were generated or analyzed during the current study.

## Abbreviations

AAPS: American Association of Pharmaceutical Scientists
AAid: Assessment Aid
BLA: Biologics License Application
ANVISA: Brazil National Health Surveillance Agency
CMC: Chemistry, Manufacturing, and Controls
EUA: Emergency Use Authorization
EMA: European Medicines Agency
FEI: FDA Establishment Identification
FDA: Food and Drug Administration
ICH: International Council for Harmonisation of Technical Requirements for Pharmaceuticals for Human Use
IND: Investigational New Drug Application
NDA: New Drug Application
NME: New molecular entity
OPMA: Office of Pharmaceutical Manufacturing Assessment
OPQ: Office of Pharmaceutical Quality
OCE: Oncology Center of Excellence
PDUFA: Prescription Drug User Fee Act
PQAAid: Product Quality Assessment Aid
POP: Project Orbis Partners
QbD: Quality by design
RTOR: Real-Time Oncology Review
HSA: Singapore Health Sciences Authority
TGA: Therapeutics Goods Administration
MHRA: United Kingdom Medicines and Healthcare Products Regulatory Agency
